# Scintigraphic Detection of Dual Ectopic Thyroid Tissue: Experience of a Chinese Tertiary Hospital

**DOI:** 10.1371/journal.pone.0095686

**Published:** 2014-04-18

**Authors:** Zhaowei Meng, Shanshan Lou, Jian Tan, Qiang Jia, Rongxiu Zheng, Geli Liu, Mei Zhu, Qing He, Dong Li

**Affiliations:** 1 Department of Nuclear Medicine, Tianjin Medical University General Hospital, Tianjin, Tianjin, P. R. China; 2 Department of Mathematics, Statistics and Printing Press, Tianjin Normal University, Tianjin, Tianjin, P. R. China; 3 Department of Pediatrics, Tianjin Medical University General Hospital, Tianjin, Tianjin, P. R. China; 4 Department of Endocrinology, Tianjin Medical University General Hospital, Tianjin, Tianjin, P. R. China; 5 Department of Radiology, Tianjin Medical University General Hospital, Tianjin, Tianjin, P. R. China; Centre for Inflammation Research, United Kingdom

## Abstract

**Purpose:**

To assess scintigraphic pattern, clinical indication and relevance of dual ectopic thyroid tissue (ETT). Literature is reviewed for such cases.

**Methods:**

In this 5-year retrospective study, we reviewed all thyroid scintigraphies in our data base. Patients diagnosed with suspected ETT were identified. Literature is reviewed. Statistics were done by one-way analysis of variance and least significant difference test.

**Results:**

From 11905 thyroid scintigraphies during the 5-year period, we retrieved 121 patients eligible for analysis. The top two indications were assessing a palpable front neck mass to determine whether it was an ETT, and primary hypothyroidism. Patients were divided into 3 groups. Group 1 with single ETT (83 cases); group 2 with dual ETT (6 cases) and group 3 with athyroid (32 cases). Age and thyroid hormones were highest in group 2, and lowest in group 3. Thyrotropin was highest in group 3, and lowest in group 2. Thyroxine was given to hypothyroid patients, while no surgery was performed. There were 42 published cases with dual ETT, most of whom were under 30 years old. 38.10% of them were euthyroid, 33.33% hypothyroid, and 21.43% subclinical hypothyroid. Most frequent ectopic positions included lingual (33.73%), sublingual (27.71%) and subhyoid (22.89%).

**Conclusions:**

In our cohort, incidence of dual ETT was 0.05% if the denominator was total number of thyroid scintigraphies. The incidence was 4.96% if the denominator was the number of patients with suspected ETT. Important clinical indication is a front neck palpable mass suggestive of an ETT. Important clinical relevance of recognizing the dual ETT pattern is to avoid inappropriate surgery. After reviewing all published cases, we find dual ETT is often seen in young patients. Most of such patients are euthyroid or mildly hypothyroid. Thyroid ectopia often resides in lingual, sublingual and subhyoid areas.

## Introduction

The thyroid gland is the first endocrine gland to develop during embryogenesis starting from approximately 24 days of fetal life. The thyroid anlage completes its descent on reaching anterior to the trachea in the lower neck in the 7th gestational week. The thyroid primordium occasionally fails to descend via the normal pathway and result in thyroid ectopia. Ectopic thyroid tissue (ETT) refers to all cases in which the thyroid gland is present at a location other than its usual site. The most commonly found ETT is at the base of the tongue, which accounts for 90% of all reported cases. ETT is a rare anomaly, yet it is further uncommon for dual ETT to be demonstrated simultaneously. In the English literature, we retrieved only 36 cases of dual ectopic thyroid so far [1], [2], [3], [4], [5], [6], [7], [8], [9], [10], [11], [12], [13], [14], [15], [16], [17], [18], [19], [20], [21], [22], [23], [24]. In the present study, we retrospectively reviewed all thyroid scintigraphies and medical documents over a time period of 5 years at our institution. We wanted to retrieve all patients diagnosed with suspected ETT, in particular dual ETT. We aimed to give some explanations to clinical indications and clinical relevance of dual ETT. We also estimated the rough incidence of dual ETT in our cohorts. And finally we characterized and analyzed dual ETT after reviewing all published cases.

## Materials and Methods

This study is a retrospective evaluation of the routinely obtained clinical data from June 1st 2008 to June 1st 2013. All patients who were referred to us for a thyroid scintigraphy were reviewed. Patients included for analysis were those diagnosed with suspected ETT. Thyroid scintigraphy was performed in order to detect the existence and location of ETT, as well as assessing the orthotopic thyroid. Imaging protocol was implemented as follows: 30 minutes after injection of 1 to 5 mCi (low doses were given to younger patients with lower body weights) ^99m^Tc-pertechnetate, thyroid scintigraphy was performed by using a high-resolution low-energy parallel-hole collimator equipped dual-detector scanner (Discovery VH; General Electric Medical Systems). Serum thyroid hormones were tested by an immunofluorometric assay, including free triiodothyronine (FT3, reference 3.50–6.50 pmol/L), free thyroxine (FT4, reference 11.50–23.50 pmol/L), and thyrotrophin (reference 0.20–5.00 µIU/mL, maximum measurement level 150 µIU/mL). Primary hypothyroidism (PH) diagnosis was made if simultaneous decrease of serum FT4 and/or FT3 and increase of thyrotropin were observed. Neck ultrasonography was performed to evaluate the orthotopic thyroid structure by using a color doppler ultrasound machine (GE Vingmed Ultrasound Vivid Five, Horten, Norway). Absent of orthotopic thyroid was first diagnosed by ultrasonography, and then confirmed by thyroid scintigraphy. Athyroid was defined as no orthotopic thyroid nor ETT. All PH patients were treated with thyroid hormone to maintain euthyroidism. All participants were invited to come to provide their written informed consent to participate in this study (for children participants who were unable to write, written consent was signed by their parents). This study and the consent procedure were approved by the Institutional Review Board of Tianjin Medical University General Hospital.

The electronic database PubMed (from 1964 to October 2013) was searched by using the keywords of ectopic thyroid or thyroid ectopia. Then papers describing dual ETT were identified.

All data were presented as mean ± SD. Statistics were performed with SPSS 17.0 (SPSS Incorporated, IL, USA). Differences between groups were analyzed by one-way analysis of variance (ANOVA). Least significant difference (LSD) test was used for multiple comparisons among groups. *P* value not exceeding 0.05 was considered statistically significant.

## Results

The data archive from our department displayed that the total case number of thyroid scintigraphy was 11905 from June 1st 2008 to June 1st 2013 (yearly number was 2209, 2240, 2471, 2480 and 2505, respectively). Tianjin Medical University General Hospital is the most comprehensive tertiary hospital in Tianjin municipality (population roughly 14 million in 2012). We retrieved 121 patients (90 females and 31 males, ratio 3∶1) eligible for analysis (who were diagnosed with suspected ETT). All of the patients were drug-naïve (who have never received any thyroid hormone treatment). Included patients were divided into 3 groups based on thyroid scintigraphic findings. There were 83 cases with single ETT (group 1), 6 cases with dual ETT (group 2) and 32 cases with athyroid (group 3). Incidence of dual ETT could be calculated as 0.05% if the denominator was the number of all patients underwent thyroid scintigraphy. The incidence could also be calculated as 4.96% if the denominator was the number of patients with suspected ETT. Clinical indications of the 121 patients were listed in **Table 1**. It was demonstrated that most of the patients had 2 indications for thyroid scintigraphy. We found that the most important two indications were 1) assessing a palpable front neck mass to determine whether it was an ETT, and 2) primary hypothyroidism.

**Table 1:** Clinical indications of the included patients in our study.

Groups*	Case number	Primary indication	Secondary indication
1	40	Assessing front neck mass	Primary hypothyroidism
	17	Assessing front neck mass	None
	13	Primary hypothyroidism	Assessing front neck mass
	10	Primary hypothyroidism	Ruling out thyroid ectopia
	3	Assessing thyroid nodule or orthotopic thyroid uptake function	Assessing front neck mass
2	4	Assessing front neck mass	None
	2	Primary hypothyroidism	Assessing front neck mass
3	32	Primary hypothyroidism	Ruling out thyroid ectopia

*group 1 = single ectopic thyroid tissue, group 2 = dual ectopic thyroid tissue, group 3 = athyroid.

There were significant differences of all clinical data among the 3 groups of patients after ANOVA analyses (**Table 2**). LSD tests showed that age and thyroid hormones in group 2 were significantly higher than any of the other 2 groups, and in addition, age and thyroid hormones in group 1 were significantly higher than group 3. However, thyrotropin was highest in group 3, and lowest in group 2 (**Table 2**). These results indicate that patients with dual ETT tend to have less severe hypothyroidism, some are euthyroid, and therefore, they will come to medical consultations much later. On the other hand, patients with athyroid have overt hypothyroidism, all of whom are identified during the neonatal screening and referred to us for a confirmation diagnosis younger than 1 year of age.

**Table 2:** Clinical data of the included patients in our study.

Groups (case number)*	Age	Free triiodothyronine (reference 3.50–6.50 pmol/L)	Free thyroxine (reference 11.50–23.50 pmol/L)	Thyrotrophin (reference 0.20–5.00 µIU/mL)
1 (83)	2.45±1.11	2.63±0.81	7.78±2.76	98.68±59.58
2 (6)	14.33±8.69	3.57±0.69	12.07±2.45	22.17±25.97
3 (32)	0.56±0.24	1.04±0.33	2.84±0.86	149.69±1.77
*F* value (*P* value)**	117.91 (<0.01)	68.71 (<0.01)	64.80 (<0.01)	21.43 (<0.01)
*P* value_(1)∶(2)_ ***	<0.01	<0.01	<0.01	<0.01
*P* value_(1)∶(3)_ ***	<0.01	<0.01	<0.01	<0.01
*P* value_(2)∶(3)_ ***	<0.01	<0.01	<0.01	<0.01

*group 1 = single ectopic thyroid tissue, group 2 = dual ectopic thyroid tissue, group 3 = athyroid;

**analyzed by one-way analysis of variance;

***analyzed by least significant difference test.

**Figure 1:**
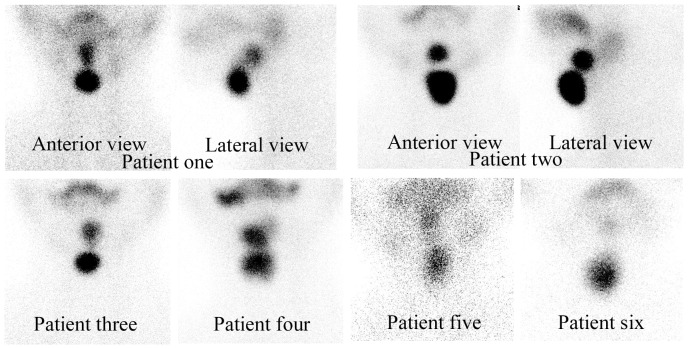
Evidence of dual ectopic thyroid tissue by thyroid scintigraphy in 6 cases in our study. Thyroid scintigraphy was performed as described in **Materials and Methods**. Dual ectopic thyroid tissue were identified in 6 cases, who displayed 2 focal areas of abnormal radiotracer uptakes in the sublingual and subhyoid regions in thyroid scintigraphies. No evidence of radiotracer uptakes in the regions of orthotopic thyroid was found. Anterior and lateral views of patient 1 and patient 2 were presented. Anterior views of the other 4 patients were shown as well.

We identified 4 girls and 2 boys with dual ETT (group 2). All of them displayed ETT in the sublingual and subhyoid regions (**Figure 1**). 2 (young adult age) of the 6 dual ETT patients were euthyroid, 2 (adolescent age) were mildly hypothyroid (1 subclinical hypothyroid), and the other 2 (toddler age) who were youngest among the dual ETT patients were hypothyroid (**Table 3**). For hypothyroid patients with dual ETT (4 cases), thyroid hormone was prescribed. For euthyroid patients with dual ETT (2 cases), close follow-up was recommended only. No surgery was performed to remove the palpable front neck ETT. We considered that the most important clinical relevance of recognizing the dual ETT pattern is to avoid inappropriate surgery.

**Table 3:** Clinical details of patients with dual ectopic thyroid tissue in our study.

Case number	Age	Sex	Free triiodothyronine (reference 3.50–6.50 pmol/L)	Free thyroxine (reference 11.50–23.50 pmol/L)	Thyrotrophin (reference 0.20–5.00 µIU/mL)
1	14	Female	3.2	10.8	8.9
2	21	Female	4.5	15.6	3.8
3	26	Female	4.3	14.5	4.2
4	16	Female	3.5	11.8	7.3
5	4	Male	2.8	9.8	65.3
6	5	Male	3.1	9.9	43.5

**Figure 2:**
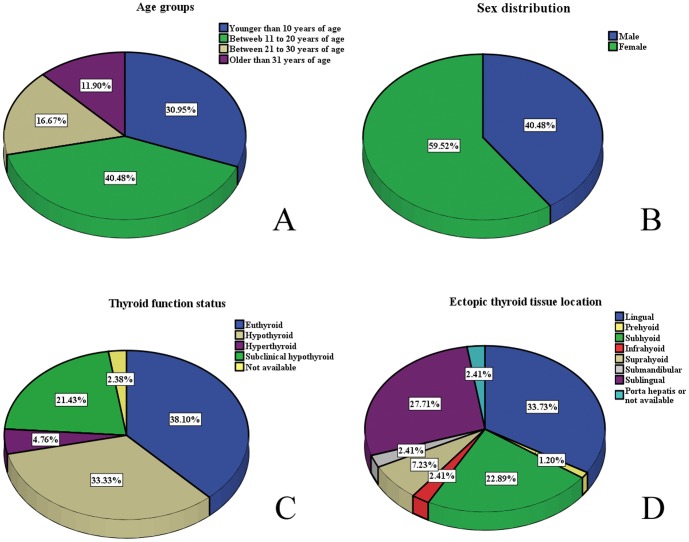
Clinical parameter distribution patterns in all reported cases with dual ectopic thyroid tissue. Altogether, clinical data of 42 cases with dual ectopic thyroid tissue were compiled. And distribution patterns of age (A), sex (B), thyroid function (C) and ectopic thyroid tissue location (D) were demonstrated in pie charts.

We retrieved and compiled all the published cases (42 patients) with dual ETT including our cases, and analyzed distribution patterns of age, sex, thyroid function and ETT locations in **Table 4** **and** **Figure 2**. We found that most of the dual ETT patients came for medical consultation when they were under 30 years of age, while 40.48% of them were between 11 to 20 years of age and 30.95% of them were younger than 10 years of age. Dual ETT affected female more frequently (59.52%) than male (40.48%). 38.10% of the patients were euthyroid, 33.33% were hypothyroid, and 21.43% were subclinical hypothyroid. Most frequent ectopic positions of ETT included lingual (33.73%), sublingual (27.71%) and subhyoid (22.89%).

**Table 4:** Summary of patients with dual ectopic thyroid tissues from literature and the current study.

Case number	Author	Age	Sex	Clinical status	Sites of ectopic thyroid
1	Long et al. [1]	14 years	Male	Euthyroid	Lingual and subhyoid
2	Kuehn et al. [2]	23 years	Female	Hyperthyroid	Lingual and subhyoid
3	Alexandre et al. [3]	28 years	Female	Not available	Lingual and subhyoid
4	Hung et al. [4]	4 years	Male	Euthyroid	Lingual and suprahyoid
5	Rosen et al. [5]	12 years	Male	Euthyroid	Lingual and subhyoid
6	Meyerowitz et al. [6]	8 years	Female	Euthyroid	Lingual and subhyoid
7	Misaki et al. [7]	4 years	Male	Subclinical hypothyroid	Lingual and sublingual
8		29 years	Female	Euthyroid	Lingual and sublingual
9	Bhatnagar et al. [8]	17 years	Female	Hypothyroid	Lingual and suprahyoid
10	Hazarika et al. [9]	32 years	Male	Euthyroid	Sublingual and suprahyoid
11		18 years	Male	Euthyroid	Sublingual and subhyoid
12	Kumar et al. [10]	14 years	Male	Euthyroid	Sublingual and subhyoid
13	Hod et al. [11]	21 years	Male	Subclinical hypothyroid	Lingual and prehyoid
14	Baik et al. [12]	15 years	Female	Euthyroid	Lingual and infrahyoid
15	Ulug et al. [13]	20 years	Female	Euthyroid	Lingual and infrahyoid
16	Ghanem et al. [14]	24 years	Female	Euthyroid	Lingual and porta hepatis
17	Al-Akeely et al. [15]	16 years	Male	Subclinical hypothyroid	Lingual and subhyoid
18	Emlik et al. [16]	43 years	Female	Euthyroid	Lingual and submandibular
19	Kisakol et al. [17]	45 years	Male	Hyperthyroid	Not available
20	Bayat et al. [18]	13 years	Female	Subclinical hypothyroid	Lingual and suprahyoid
21		13 years	Female	Subclinical hypothyroid	Lingual and suprahyoid
22	Basu et al. [19]	11 years	Female	Hypothyroid	Sublingual and subhyoid
23	Chawala et al. [20]	14 years	Male	Subclinical hypothyroid	Sublingual and subhyoid
24		12 years	Male	Hypothyroid	Sublingual and suprahyoid
25		11 years	Female	Hypothyroid	Lingual and subhyoid
26		11 years	Female	Subclinical hypothyroid	Lingual and subhyoid
27	Huang et al. [21]	71 years	Female	Euthyroid	Lingual and submandibular
28	Sood et al. [22]	7 years	Female	Subclinical hypothyroid	Sublingual and subhyoid
29	Kwon et al. [23]	37 years	Female	Euthyroid	Lingual and sublingual
30	Wildi-Runge et al. [24]	11 days	Female	Hypothyroid	Lingual and sublingual
31		20 days	Female	Hypothyroid	Lingual and sublingual
32		42 days	Male	Hypothyroid	Lingual and sublingual
33		13 days	Male	Hypothyroid	Lingual and sublingual
34		13 days	Female	Hypothyroid	Lingual and sublingual
35		11 days	Female	Hypothyroid	Lingual and sublingual
36		19 days	Male	Hypothyroid	Lingual and sublingual
37	Current report	14 years	Female	Hypothyroid	Sublingual and subhyoid
38		21 years	Female	Euthyroid	Sublingual and subhyoid
39		26 years	Female	Euthyroid	Sublingual and subhyoid
40		16 years	Female	Subclinical hypothyroid	Sublingual and subhyoid
41		4 years	Male	Hypothyroid	Sublingual and subhyoid
42		5 years	Male	Hypothyroid	Sublingual and subhyoid

## Discussion

ETT is an uncommon congenital aberration resulting from an abnormal migration of the thyroid gland. It is very unusual for two ectopic foci to be present simultaneously, the exact incidence of which is still not known. Wildi-Runge et al. [24] used thyroid scintigraphy during the initial diagnostic procedure for neonate PH patients, and found 9% incidence. The denominator for this high incidence was neonates with congenital hypothyroidism. Our series, which is the largest till now, showed an incidence of 0.05% if the denominator was the number of all patients underwent thyroid scintigraphy. If the denominator was the number of patients with suspected ETT, the incidence was 4.96%. We consider that the actual incidence of dual ETT may be largely underestimated due to two main reasons. First, there are only scant reports of individual cases, except for Wildi-Runge's study and the current study, no other large series have been documented. Second, there must be a number of asymptomatic patients with euthyroidism who will never come for medical consultation. On reviewing all the cases already published, including the present investigation, it is apparent that patients with dual ETT are likely to be euthyroid or less severe hypothyroid, which can explain why they have delayed needs for seeking medical help. In fact, we also found that ages of patients with dual ETT were significantly higher than other patients without orthotopic thyroid. Our study demonstrated that mean thyroid hormones of patients with dual ETT were even within normal levels, and thyrotropin levels were significantly lower than any of the other 2 patient groups. These features of dual ETT were consistent with previous reports with reviews [20], [22], [24].

Embryonic mechanism for dual ETT remains elusive. The molecular pathology of thyroid ectopia in mouse models has demonstrated that the Foxe 1 mutation is necessary for thyroid migration [25]. And mutations in thyroid transcription factors TTF-1, TTF-2 and PAX-8 have been implicated in thyroid dysgenesis and abnormal migration of the thyroid bud [26]. However, to date, no gene has been confirmed to be associated with the human thyroid ectopia [22], [24], [25]. At least two postulations may help to explain human dual ETT phenomenon [24], [27], [28]. First, insufficient signaling gradients in surrounding tissues might lead to two diverging migration profiles of the thyroid anlage at an early stage of organogenesis, which would result in dual ectopy [27], [28], [29]. Second, migration defects from indirect indication of polyclonality or oligoclonality of primordial thyroid cells will give them no potential to reach the normal location and then to develop into a bilobed gland [29]. It is reported that the thyroid gland is indeed derived from a few cell clones, at least perhaps two clones (one for each lobe) [30]. In normal thyroid tissue, this has no impact on organogenesis; nevertheless, in a dysgenetic thyroid two populations of thyroid follicular cell precursors with different genomic architecture can have different inherent migration capacity [31].

Diagnosis and treatment of dual ETT rely on thyroid scintigraphy profoundly, which can explain the clincal relevance of this nuclear medicine imaging procedure. Thyroid scintigraphy is the most reliable method to recognize all sites of functioning ETT and to confirm absence of the orthotopic thyroid. Much more importantly, it is both sensitive and specific for the differentiation of a front neck ETT from other causes of midline cervical masses such as thyroglossal duct cyst, lipoma, enlarged lymph node, epidermoid cyst, vascular malformation and malignancies. It can also differentiate between a lingual ETT and other swellings in the base of the tongue such as hypertrophic lingual tonsil, vallecular cyst, and mucous retention cyst [20], [22]. From our experience, assessing front neck mass to differentiate ETT from other lesions is the most important clinical indication for thyroid scintigraphy. Once the dual ETT diagnosis is established, surgical removal of the thyroid ectopia is not appropriate in most of the time, as they may be the only functioning thyroid tissues in the body. For example, for the currently reported 6 cases, we did not implement any invasive intervention. Instead, thyroid hormone replacement was prescribed for hypothyroid patients with dual ETT. In fact, early diagnosis is also essential for the proper pharmacological management at an early stage, since PH in neonates or young infants may lead to retarded growth and psychoneurological development [32]. For euthyroid patients with dual ETT, close follow-up is recommended.

In conclusion, dual ETT is a rare entity, which is often discovered in young and adolescent patients. Most of these patients are with mildly altered or without altered thyroid metabolic status. Dual ETT often resides in positions like lingual, sublingual and subhyoid. The present investigation and the published literatures underline the clinical value of thyroid scintigraphy, which should be performed in any patient with a clinical indication that a front neck palpable mass is suspected to be a ETT. The most important clinical relevance of recognizing the dual ETT pattern in thyroid scintigraphy is to prevent inappropriate excision.
